# Millisecond time-resolved serial oscillation crystallography of a blue-light photoreceptor at a synchrotron

**DOI:** 10.1107/S2052252520007411

**Published:** 2020-06-24

**Authors:** Sylvain Aumonier, Gianluca Santoni, Guillaume Gotthard, David von Stetten, Gordon A. Leonard, Antoine Royant

**Affiliations:** a Structural Biology Group, European Synchrotron Radiation Facility, 71 avenue des Martyrs, Grenoble Cedex 9, 38043, France; b Institut de Biologie Structurale (IBS), Université Grenoble Alpes, CEA, CNRS, 71 avenue des Martyrs, Grenoble Cedex 9, 38044, France

**Keywords:** room-temperature oscillation X-ray crystallography, time-resolved crystallography, serial crystallography, plant photoreceptors

## Abstract

The recent development of serial crystallography has popularized time-resolved crystallography as a technique to determine the structure of protein-reaction intermediate states. However, most approaches rely on the availability of thousands to millions of microcrystals. A method is reported here, using monochromatic synchrotron radiation, for the room-temperature collection, processing and merging of X-ray oscillation diffraction data from <100 samples in order to observe the build up of a photoreaction intermediate species. Using this method, we monitored with a time resolution of 63 ms how the population of a blue-light photoreceptor domain in a crystal progressively photoconverts from the dark to the light state. The series of resulting snapshots allows us to visualize in detail the gradual rearrangement of both the protein and chromophore during this process.

## Abstract 2

A method is presented to perform time-resolved X-ray crystallography with a 63 ms time resolution using a fast pixel detector and partial oscillation data sets. This minimizes the number of crystals (<100) required for a complete experiment.

## 1. Introduction   

Synchrotron-based X-ray crystallography has been largely responsible for the explosion in macromolecular crystallography, which has occurred since the early 2000s (http://biosync.sbkb.org) and during which the overwhelming fraction of the structural information was obtained by exploiting crystals in which the conformation and biological state of the system under study is static, whether held at cryogenic or room temperature. However, gaining insight into the molecular mechanism of a protein greatly benefits from watching it in action [*i.e.* while it is performing its function (Hajdu *et al.*, 2000 ▸; Bourgeois & Royant, 2005 ▸; Šrajer & Schmidt, 2017 ▸)] and, for this, time-resolved crystallography (TRX) experiments are necessary. Room-temperature Laue crystallography pioneered TRX at synchrotrons, providing a time resolution down to the 100 ps level (Schotte *et al.*, 2003 ▸). However, the number of systems to which the method could be applied is limited. Thus, although the technique produced some impressive successes (Wöhri *et al.*, 2010 ▸; Schotte *et al.*, 2012 ▸, 2003 ▸), TRX gradually fell out of favour. Recently, the development of serial crystallography (SX) (Chapman *et al.*, 2011 ▸), coupled with advances in sample delivery (Grünbein & Kovacs, 2019 ▸) and X-ray detectors (Broennimann *et al.*, 2006 ▸; Casanas *et al.*, 2016 ▸), has resulted in a renewed interest in TRX, first at free-electron lasers (TR-SFX, time-resolved serial femtosecond crystallography) (Barends *et al.*, 2015 ▸; Pande *et al.*, 2016 ▸; Nango *et al.*, 2016 ▸; Kern *et al.*, 2018 ▸), then at synchrotron sources (TR-SSX, time-resolved serial synchrotron crystallography) (Schulz *et al.*, 2018 ▸; Mehrabi, Schulz, Dsouza *et al.*, 2019 ▸; Mehrabi, Schulz, Agthe *et al.*, 2019 ▸). However, both TR-SFX and TR-SSX techniques are, in the main, currently based on the recording of still diffraction images from many thousands of crystals for each time point, and their requirement in sample quantity is thus very high.

**Figure 1:**
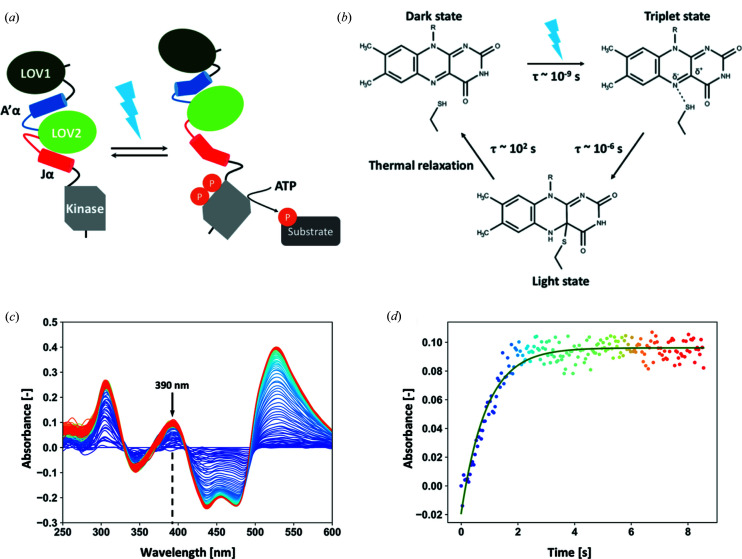
Photoactivation of the LOV2 domain of Phot2 from *A. thaliana.* (*a*) Schematic of Phot2 domain reorganization upon blue-light illumination (adapted from the work of Okajima *et al.*, 2014 ▸). (*b*) The photocycle of *At*Phot2LOV2. (*c*) Time-resolved series of UV–Vis absorption difference spectra of a *At*Phot2LOV2 crystal under 3.2 µW irradiation of a 470 nm LED, recorded at 23 Hz [colour range: violet (*t* = 0 s) to red (*t* = 9 s)]. The absorption maximum of the light state is indicated with an arrow. We note that the apparent increase in absorbance above 500 nm is caused by the decrease in dark-state blue-light-induced fluorescence. (*d*) Evolution of the optical density at λ = 390 nm, which is characteristic of the *At*Phot2LOV2 light-state build up (rise time τ = 0.891 s).

As an alternative to TR-SSX, we have developed a method, which we called time-resolved serial oscillation crystallography (TR-SOX), that exploits diffraction data collected from only tens to hundreds of crystals and has the potential to produce millisecond time-resolution movies of biomolecular processes occurring in crystals. TR-SOX was used to probe the build up of the light state in the photocycle of the LOV2 domain of Phot2 (phototropin-2) from *Arabidopsis thaliana* (*At*Phot2LOV2) with a time resolution of 63 ms. Phot2 is a high-intensity blue-light photoreceptor mediating phototropism in higher plants (Christie, 2007 ▸). The protein comprises two successive light–oxygen–voltage (LOV) domains (LOV1 and LOV2) separated by the α-helix A′α, and a kinase domain separated from LOV2 by the α-helix Jα [Fig. 1 ▸(*a*)]. Through their connection to the LOV1 and kinase domains, both α-helices can play a role in photoreceptor dimerization, prolongation of the photoreceptor signalling state duration or signal transduction (Halavaty & Moffat, 2013 ▸; Okajima *et al.*, 2014 ▸). In particular, Jα unfolds upon blue-light irradiation, triggering kinase activity (Harper *et al.*, 2003 ▸). The photoactivation of LOV domains has been intensively studied by various biophysical methods including fast spectroscopic techniques (Swartz *et al.*, 2001 ▸; Kottke *et al.*, 2003 ▸; Kennis *et al.*, 2003 ▸). The dark state of *At*Phot2LOV2 absorbs light maximally at λ = 447 nm via its flavin mononucleotide (FMN) chromophore. Upon photon absorption, a triplet state absorbing at λ = 660 nm forms in nanoseconds, which then converts on the microsecond time scale to the light state, a covalent flavin–cysteinyl adduct absorbing at λ = 390 nm [Fig. 1 ▸(*b*)]. Finally, the system relaxes back to the dark state on the 100 s level.

## 2. Methods   

### 2.1. Protein expression and purification   

The gene coding for the Phot2 LOV2 domain from *A. thaliana* (*At*Phot2LOV2) with a C-terminal hexahistidine tag was synthetized (Genecust, Ellange, Luxemburg) and inserted in a pBad vector using BamHI/PmeI cloning sites. The vector was transformed into the *Escherichia coli* host strain BL21 (Invitrogen) and grown on ampicillin (100 µg ml^−1^) selective agar plates. Cells were grown in 2YT medium supplemented with ampicillin (100 µg ml^−1^) at 37°C until an optical density of 0.6 at 600 nm was reached. Protein expression was then induced with 0.02% arabinose and cells were grown overnight at 17°C. Cells were harvested and centrifuged at 4000*g* for 20 min at 4°C. Pellets were resuspended in 25 ml of lysis buffer [50 m*M* Tris pH 8.0, 300 m*M* NaCl, 10 m*M* imidazole, 0.25 mg ml^−1^ lysozyme, 400 µg ml^−1^ DNAse I, 20 m*M* MgSO_4_, 1 tablet of the EDTA-free protease inhibitor cocktail cOmplete (Roche, Basel, Switzerland)] per litre of centrifuged medium and frozen at −80°C. Thawed pellets were sonicated and cell debris was centrifuged at 15 000*g*, for 45 min at 4°C. The protein was purified from the clarified lysate using a nickel affinity column (His-Trap HP 5 ml, GE HealthCare) and eluted against an imidazole gradient (50 m*M* Tris pH 8.0, 300 m*M* NaCl, 10–500 m*M* imidazole). A second purification step consisted of size-exclusion chromatography (Superdex 75 10/300 GL, GE HealthCare) in a 20 m*M* Tris pH 8.0 buffer, after which the purified protein was concentrated to 8 mg ml^−1^.

### 2.2. Protein crystallization   

Prior to crystallization, *At*Phot2LOV2 aliquots were submitted to limited proteolysis using 0.5 µg µl^−1^ trypsin, which had been found to be crucial in controlling crystal quality. A first batch of crystals were produced by the hanging-drop vapour diffusion method (2 µl drops of 1:1, mother liquor:protein ratio) in a condition consisting of 12–17% PEG8000, 200 m*M* calcium acetate in 100 m*M* MES buffer pH 6.0. This resulted in square-shaped crystals of heterogeneous size, which were then used for microseeding with a 1:100 dilution of the seed solution with the protein solution. The resulting crystals are of a much more homogeneous size of 50 × 50 × 50 µm.

### 2.3. *In crystallo* UV–visible absorption spectroscopy   

UV–vis absorption spectra of *At*Phot2LOV2 were obtained at the *ic*OS Laboratory at the European Synchrotron Radiation Facility (ESRF) (von Stetten *et al.*, 2015 ▸). The reference light is provided by a DH2000-BAL lamp (Ocean Optics, Largo, Florida) and connected to the setup by a 200 µm optical fibre. The transmitted light is connected to a grating-based QE65Pro spectrophotometer (Ocean Optics, Largo, Florida) via a 400 µm fibre to the output detector. The actinic light is output by a light-emitting diode (LED) of peak wavelength λ = 470 nm (Model M470F3, Thorlabs, Newton, New Jersey) connected to the setup via a 1000 µm fibre. The tuneable power of the LED was checked with an energy meter (Nova II, Ophir, Jerusalem, Israel) in the range 0–27.5 mW. The resulting power on the crystal was thus varied between 0 and 3.2 µW.

### 2.4. Diffraction data collection and sorting   

All diffraction data were collected using ESRF beamline ID30A-3 (Theveneau *et al.*, 2013 ▸; von Stetten *et al.*, 2020 ▸). Each crystal was maintained at room temperature using a humidity controller (HC1, Arinax, Voreppe, France) with a relative humidity set at 99.65% [value determined from the composition of the mother liquor (Wheeler *et al.*, 2012 ▸)]. The LED output was collimated at the entrance of a 5× magnifying objective positioned 75 mm from the sample position so as to deliver 3.5 µW to the crystal (see Figs. S1 and S2 in the Supporting information). The limited illumination power ensures a gradual slow photo-conversion of the whole crystal from the top right corner (point of the illumination) to the bottom left corner (see Video S1 in the Supporting information).

Each crystal was manually mounted on the MD2 microdiffractometer (Arinax, Voreppe, France) with the top right corner of the crystal centred, to our best efforts, in the 15 µm diameter X-ray beam. Each data collection comprised 1000 images of 0.5° oscillation collected with an exposure time of 4.2 ms. For each data set the first 990 images were binned into 66 × 15-image sub-data sets, corresponding to 66 time points. In order to prevent significant resolution loss during data collection caused by global radiation damage, the X-ray flux was limited to between 4.4 × 10^10^ and 5.7 × 10^10^ photons s^−1^, corresponding to an absorbed dose of between 115 and 162 kGy per full data collection. We performed 88 such data collections to monitor the build up of the light state. The LED was triggered by the rising edge of the TTL (transistor–transistor logic) signal used to trigger the EIGER X 4M detector, which signals the start of the data collection synchronized with X-ray shutter opening and goniometer rotation. In order to generate the reference dark-state structure, we repeated a similar experiment on 32 different crystals without LED illumination.

### 2.5. Diffraction data set processing   

All of the crystals belonged to the space group *P*4_3_2_1_2. For each of the 88 light-state data sets, and in anticipation of the situation where all 15-image sub-wedges would come from independent crystals thus allowing each crystal to absorb a significantly higher X-ray dose, each 15-image sub-wedge was separately integrated using *XDS* (Kabsch, 2010 ▸). The 66 sub-wedges of each of the 88 data sets corresponding to the 66 time points were then subjected to analysis using *ccCluster* (Santoni *et al.*, 2017 ▸), which clusters isomorphous sub-data sets and eliminates outliers. Based on the output of the program, suitable clusters of sub-wedges were carried forward to the next stage. Here, reflections were combined into a single file using *POINTLESS* (Evans, 2011 ▸) and symmetry-related reflections were then scaled and merged using *AIMLESS* (Evans & Murshudov, 2013 ▸). Final resolution limits were assigned using a CC_1/2_ cutoff (Karplus & Diederichs, 2012 ▸) of 0.70 in the highest-resolution shell with 〈*I*/σ(*I*)〉 greater than 1.0. Inclusion of clustering nodes with increasing distance cutoffs was iteratively tested until the diffraction resolution, as defined above, could be maximized. In order to obtain a high-quality reference data set, the dark-state data set was composed by merging the images of the best cluster (composed of five data sets) obtained from the 32 data sets (restricted to their first 500 images, as they provided a sufficient quantity of data) recorded in the absence of LED illumination.

### 2.6. Structure determination and refinement of the dark and light states   

A model for the dark-state data set (time point *t* < 0 ms) was constructed from a previously determined room-temperature structure of *At*Phot2LOV2 (see the PDB entry 6qqj) (Gotthard *et al.*, 2019 ▸). Structure refinement (including the four parameters *x*, *y*, *z* and *B* for each atom and group occupancy for each residue in alternate conformation) was performed using *Phenix* (Adams *et al.*, 2010 ▸) with iterative inspection of electron-density maps in *Coot* (Emsley *et al.*, 2010 ▸). This led to the identification of ten residues in alternative conformations A and B, including Cys426. The model consists of residues 388 to 492, Glu388 being the second residue before the first strand and Gly492 being the fourth residue after the fifth and final strand of the LOV2 domain. There is an additional me­thio­nine at the N terminus, added for expression purposes, and ten residues of the linker forming a short five-residue helix at the C terminus.

A model for the light state was obtained using the data set at time point *t*_66_ = 4158 ms. Initial maps were calculated by inserting the fixed dark-state structure determined at *t* < 0 ms. Residual densities in *F*_obs_ − *F*_calc_ and 2*F*_obs_ − *F*_calc_ maps were investigated, and checked in maps at earlier time points 60 to 65 for consistency. A number of residues showed clear displacements and were modelled as alternate conformation C. In order to allow for the accommodation of significant movements of the main chain, residues flanking those in alternate conformation C were also modelled as such. Overall, six continuous stretches of the protein sequence were modelled in alternate conformations A and C. The two key residues Cys426 and Phe470 were modelled in three conformations: Cys426A, Cys426B and Phe470A in the dark state; and Cys426C, Phe470C and Phe470D in the light state. For consistency of occupancy refinement, the ten residues with a conformer B in those stretches have been limited to their A conformer in the fixed dark state. Once a satisfying atomic model of the light state had been obtained, occupancies for each of the six strands were refined. The structures of the dark state (time point *t*_0_ = 0 ms) and light state (time point *t*_66_ = 4158 ms) of *At*Phot2LOV2 have been deposited in the PDB under entries 6s45 and 6s46.

### 2.7. Occupancy refinement for all time points   

Structure refinement of each time point between *t*_1_ = 63 and *t*_65_ = 4095 ms was performed by inserting a combination of the dark and light states with occupancies 0.5/0.5 for conformation A and C, and 0.33/0.33/0.33 for conformations A/B/C or A/C/D. The only parameters that were subsequently refined were the occupancies of protein segments 1 to 6.

## 3. Results   

In order to observe the phenomenon on the millisecond to second time scale, we slowed down the rate of light-state build-up within a crystal of *At*Phot2LOV2 using an attenuated 470 nm LED as actinic light. Depending on the level of attenuation, the limited number of photons photo-activate all protein molecules within the crystal in a few seconds (Video S1) to tens of seconds. The probed phenomenon is thus not the build up of the light state (which occurs in microseconds at the single-molecule level), but that of its population within the crystal. We used *in crystallo* UV–vis absorption spectroscopy at the IBS/ESRF *ic*OS Laboratory (von Stetten *et al.*, 2015 ▸) to quantify this phenomenon as a function of actinic light power. Absorption spectra were continuously recorded at a 23 Hz rate providing a 43 ms time resolution. Build-up rates were observed to be linearly correlated with LED power over a 50-fold power range (Fig. S1), proving that light-state build up in the crystal is solely accounted for by the number of photons impinging on the crystal. We thus determined that a light power of 3.2 µW at the crystal surface would result in a build-up time constant just below 1 s (891 ms) [Figs. 1 ▸(*c*) and 1 ▸(*d*)].

**Figure 2:**
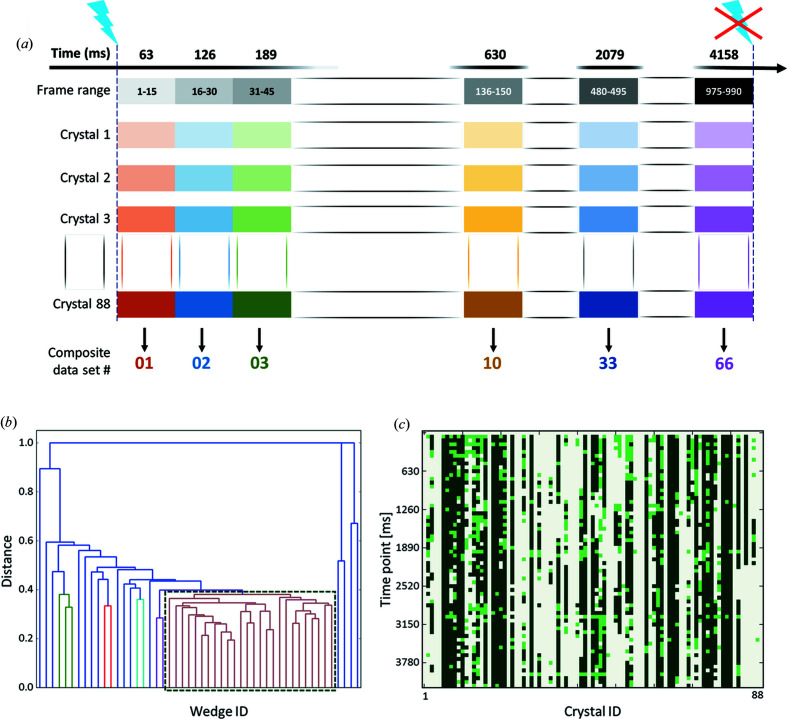
Data-collection strategy and data set composition. (*a*) Complete data sets were collected from 88 different crystals. For each crystal, the diffraction images were separated into 15 frame wedges corresponding to 66 time points (from 63 to 4158 ms). (*b*) A dendrogram of wedge clustering for the initial time point (*t* = 0–63 ms). The 26 wedges forming the cluster highlighted with a dark green rectangle group at a linkage distance of 0.39 were subsequently merged together to produce a 2.45 Å resolution data set with 100% completeness and a multiplicity of 12.6 (Table S1). (*c*) A visualization of the data set wedges integrated (light and dark green) and of the wedges retained in the composite data sets corresponding to the 66 different time points (dark green only).

For TR-SOX experiments on the ESRF beamline ID30A-3, 88 randomly-oriented crystals of *At*Phot2LOV2 were sequentially mounted in a wet air stream at ambient temperature, under low ambient light conditions with LED illumination synchronized with X-ray shutter opening, goniometer axis rotation and X-ray detector acquisition. The X-ray beam flux was attenuated such that the total absorbed dose for each data set was between 115 and 162 kGy, thus minimizing resolution loss and thio-ether bond reduction (Gotthard *et al.*, 2019 ▸). For each crystal, 990 images of 0.5° oscillation range were recorded at an acquisition rate of 250 Hz. Each of the 88 resulting data sets was then split into 15-frame sub-wedges assigned to 66 different time periods *T*_1_ (0–63 ms) to *T*_66_ (4095–4158 ms), which were ascribed to 66 time points from *t*_1_ = 63 ms to *t*_66_ = 4158 ms [Fig. 2 ▸(*a*)].

**Figure 3:**
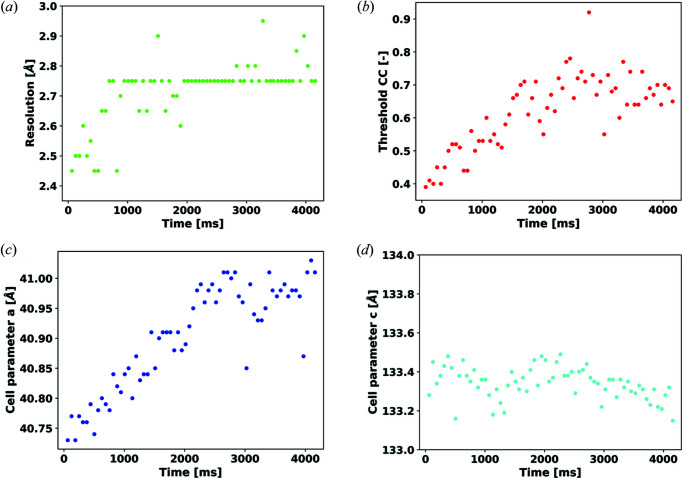
The evolution of various crystallographic data reduction parameters as a function of time. The evolution of (*a*) diffraction resolution, (*b*) distance threshold CC_threshold_ for the clustering of sub-data sets, (*c*) unit-cell parameter *a* and (*d*) unit-cell parameter *c*.

For each time point of the diffraction experiment, between 36 and 52 sub-wedges were successfully integrated and merged together using *ccCluster* (Santoni *et al.*, 2017 ▸) [Figs. 2 ▸(*b*) and 2 ▸(*c*)]. The clustering threshold CC_threshold_ was chosen such that the diffraction resolution of each final data set was maximized (see *Methods* [Sec sec2]). We thus obtained 66 complete data sets corresponding to time points *t*_1_ to *t*_66_ at a diffraction resolution starting at 2.45 Å for *t*_1_ and rapidly settling at 2.75 Å after *t*_11_ = 693 ms [see Table S1 in the Supporting information and Fig. 3 ▸(*a*)]. In addition to the final data set resolution, two other data integration and reduction parameters were also observed to evolve with time in a similar manner. First, the value CC_threshold_ used to maximize resolution diffraction increases steadily from 0.39 at *t*_1_ and stabilizes around 0.68 after *t*_24_ = 1512 ms [Fig. 3 ▸(*b*)]; second, the cell parameter *a* follows a comparable pattern by increasing from 40.73 Å at *t*_1_ to an average of 40.97 Å after *t*_34_ = 2142 ms, while the cell parameter *c* appears to remain constant within the noise [Figs. 3 ▸(*c*) and 3 ▸(*d*)]. The fact that these three parameters settle to a plateau may indicate that their evolution is not primarily driven by X-ray radiation damage but is potentially an effect of photo-activation of the protein leading to subtle changes in the unit-cell parameters and crystal contacts and thus to a decrease, as a function of time, in the degree of isomorphism.

**Figure 4:**
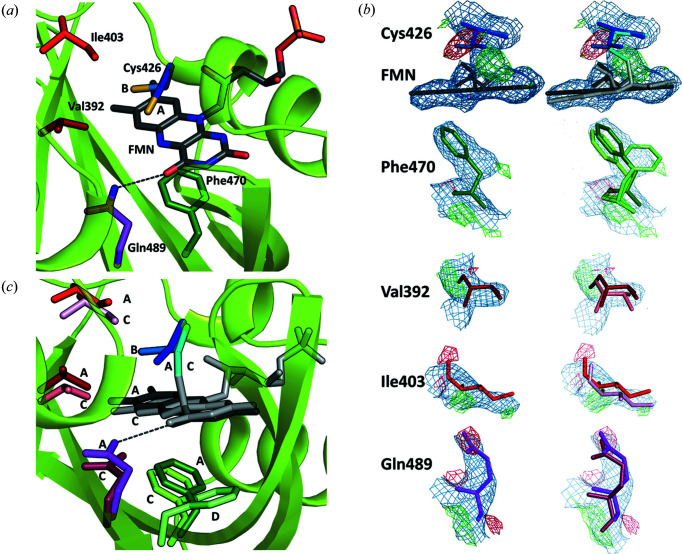
Model building of the structure of the light state of *At*Phot2LOV2. (*a*) The structure of *At*Phot2LOV2 in the dark state showing key residues surrounding the FMN chromophore. (*b*) *F*_obs_ − *F*_calc_ (green, positive; red, negative) and 2*F*_obs_ − *F*_calc_ electron-density maps, contoured at 2.5σ and 1.0σ levels, respectively, for all key residues and the FMN chromophore at time point 66 before modelling of the light state. The dark-state model (left) and both dark- and light-state models (right) are represented. (*c*) The structure of *At*Phot2LOV2 in the light state (C and D conformers, light shade colours) superimposed on that of the dark state (A and B conformers, darker shade colours).

To determine the structure of the dark state of *At*Phot2LOV2, representative of the time series before *t* = 0 ms, we carried out the same diffraction experiment, but without illumination, on a series of 32 crystals. The first 500 images of 15 data sets could be successfully integrated and were subjected to clustering with *ccCluster* which retained 5 sub-wedges using a CC_threshold_ of 0.43 and resulted in a complete highly redundant data set at 2.2 Å resolution (Table S1). Refinement of the crystal-state structure of the dark state was then carried out using a previously determined structure of *At*Phot2LOV2 (PDB entry 6qqj) (Gotthard *et al.*, 2019 ▸) as a starting model. Most residues were modelled in a single conformation A, while ten were modelled with alternate conformations A and B, including Cys426 which forms a covalent bond with the FMN in the light state. The hydro­philic side of the FMN isoalloxazine ring is stabilized by two hydrogen bonds to Asn458 and one key hydrogen bond to Gln489 [Fig. 4 ▸(*a*)].

**Figure 5:**
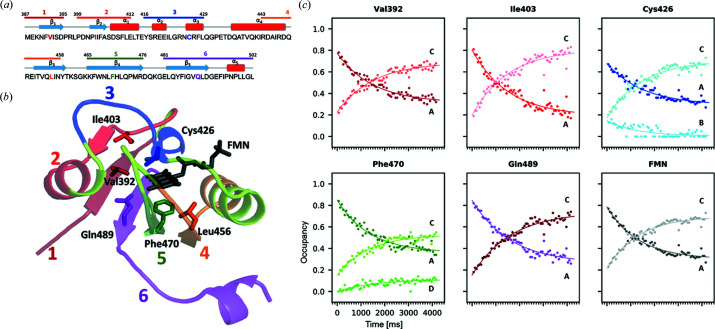
Time evolution of the relative occupancies of key residues of *At*Phot2LOV2 upon blue-light illumination. (*a*) Sequence and (*b*) structure featuring key residues in colour, secondary structure elements (α-helices and β-strands) and protein segments 1 to 6 that move between the dark and the light state. (*c*) Occupancies of the different conformations (A, B: dark state; C, D: light state) are modelled as mono-exponential rises or decays. For every residue and the cofactor, occupancy and time scales are as shown for Phe470.

Modelling of the crystal structure of the light state of *At*Phot2LOV2 [denoted C and D in Fig. 4 ▸(*c*)] was guided by inspection of *F*_obs_ − *F*_calc_ and 2*F*_obs_ − *F*_calc_ electron-density maps for the later time points (*t*_60_ to *t*_66_) in our diffraction data series. Here, in addition to the formation of the covalent bond between Cys426 and FMN and the rotation of the FMN moiety, several residues surrounding the chromophore show clear signs of side-chain translation (Gln489, Ile403, Val392), rotation and reorientation (Phe470) as well as displacement of their main-chain atoms [Fig. 4 ▸(*b*)]. Previous structures of the light state of LOV domains, in particular those described in the work of Halavaty & Moffat (2007 ▸), show a flipping of the head group of Gln489. However, in our study this flipping is not supported by atomic *B*-factor refinement, although this may be a result of a more dynamic disorder that we are unable to properly model. Phe470 is shown to adopt two new conformations C and D, the latter of which is made possible by the displacement and reorientation of Leu456 (Fig. S3). We modelled continuous sequence stretches around those residues and refined a conformation C of the protein for each of the resulting segments 1 to 6, all composed of rigid secondary-structure elements [Figs. 5 ▸(*a*), 5 ▸(*b*) and S4].

In order to follow the time course of the dark- to light-state conversion, we evaluated their respective structural contributions to each time point. Occupancies of the six segments were refined independently, while atomic coordinates and *B* factors were fixed. The evolution of the respective occupancies of each of the prominent residues and cofactor are represented in Fig. 5 ▸(*c*). All decays (conformations A and B) and rises (conformations C and D) could be modelled by a mono-exponential behaviour, whose time constants are very similar, with an average value of 1445 ± 135 ms (Table S2). This is in rather good agreement with the spectroscopy-derived rise time of 891 ms for light-state build up under similar experimental conditions [Fig. 1 ▸(*d*)]. Yet, there is a discrepancy between the maximum occupancy of the light state as observed by spectroscopy (∼100%) and that observed by X-ray crystallography (∼70%). We cannot exclude that a small fraction of the flavin–cysteinyl adduct may be reduced by X-rays but the probable explanation for the discrepancy must reside in our alternate-conformer refinement strategy, which may overestimate the proportion of the dark-state conformer at this resolution, whose structure is, overall, very close to that of the light state. Nevertheless, our results demonstrate that we have been able to visualize at the near-atomic scale the progressive conversion from the dark to the light state of *At*Phot2LOV2 molecules within the crystal on an ∼60 ms time scale.

## 4. Conclusions   

**Figure 6:**
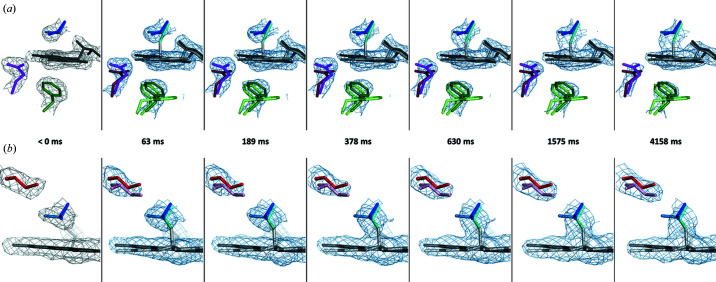
Snapshots of the molecular movie resulting from the TR-SOX experiment. (*a*) Snapshots at time points 0, 1, 3, 6, 10, 25 and 66 showing 2*F*_obs_ − *F*_calc_ electron-density maps contoured at a 1.2σ level superimposed on the structural models of the dark and light states highlighting covalent bond formation between Cys426 and the FMN, disordering of the Gln489 side chain, and displacement of the Phe470 side chain. (*b*) A different view showing 2*F*_obs_ − *F*_calc_ electron-density maps contoured at a 0.7σ level illustrating rotation of the isoalloxazine ring of the FMN, translation of Ile403 and progressive disappearance of the B conformation of Cys426.

The evolution of 2*F*_obs_ − *F*_calc_ electron-density maps and structural models obtained [Figs. 6 ▸(*a*) and 6 ▸(*b*) and Videos S2 and S3] allows an atomic description of the dark- to light-state conversion of *At*Phot2LOV2 to be determined. Absorption of a photon by FMN leads to the recruitment of both the A and B conformers of Cys426 in segment 3 to establish a covalent bond between its S_γ_ atom and the C_4a_ atom of FMN. This results in an ∼6° rotation of the chromophore isoalloxazine ring around the axis of its ribityl tail and in the disruption of a stabilizing hydrogen to Gln489. The disappearance of conformer B of Cys426 added to the rotation of the isoalloxazine ring creates a void that is readily occupied by both Val392 and Ile403 whose side chains translate by 0.6–1.1 Å towards the FMN, displacing both protein segments 1 and 2. The rotation of the chromophore pushes Phe470 into two new conformers, thus displacing segment 5. The minor conformer D of Phe470 forces a rearrangement of Leu456, thus displac­ing segment 4. Finally, the change in electronic configuration of the FMN upon covalent bond formation induces the loss of the stabilizing hydrogen bond between the FMN and Gln489, whose main and side chains move away, displacing segment 6. Segments 1 and 6, constituting the N and C termini of the protein, respectively, have already been shown to be involved in signal transduction through the modification of their interactions with the A′α and Jα helices (Halavaty & Moffat, 2007 ▸). In particular, the structure of the LOV2-Jα domain of *Avena sativa* (PDB entry 2v1a; Halavaty & Moffat, 2007 ▸) reveals that the equivalent of segments 1, 5 and 6 defined here form a groove accommodating the Jα helix and support the notion that their concerted displacement would destabilize this helix, which in turn would activate the kinase domain.

Here we have used room-temperature TR-SOX coupled with complementary *in crystallo* optical spectroscopy to follow, and provide structural insight into, the activation mechanism of a light-activated phototropin. One noteworthy advantage of this method, which is based on serial oscillation crystallography, is the significant reduction of sample material required compared with still-image-based crystallography. Obviously, our study has benefited from the high symmetry of the *At*Phot2LOV2 crystal point group, and an *n*-fold increase in the number of crystals of another biological system which produces crystals with a point group of *n*-fold lower symmetry would have to be used in order to achieve the same time resolution, with the same diffraction resolution. We have used a conservative approach by limiting the absorbed dose to ∼170 kGy for a complete data set on a single crystal, thus limiting the diffraction resolution of the reconstructed data sets. A straightforward extension of the method would be to record fewer sub-wedges from more individual crystals, thus allowing each crystal to absorb a higher dose, up to 66 times more, maximizing the resolution of resulting data sets. The method could be extended to non-photoactivable proteins by using microfluidics-based ‘mix-and-diffuse’ techniques, which would allow for pH jumps or the diffusion of substrates or ligands. Besides, we have shown that limiting the photon flux to the sample slows down the build up of the population of a sub-millisecond intermediate state from an initial state, which provides the opportunity to visualize the progressive conversion from one state to the other with a redundant set of information. In this study, the time resolution obtained was limited by a maximum goniometer rotation speed (180° s^−1^) and detector readout rate (250 Hz). Maximizing both of these aspects will probably lead to recording molecular movies on the sub-millisecond time scale. This will allow researchers to characterize large-scale structural changes that are often associated with photoreceptor or photoenzyme activation, providing an alternative to XFEL experiments for microsecond to millisecond protein dynamics studies.

## Supplementary Material

Supporting figures and video captions. DOI: 10.1107/S2052252520007411/mf5042sup1.pdf

Video S1. DOI: 10.1107/S2052252520007411/mf5042sup2.mp4

Video S2. DOI: 10.1107/S2052252520007411/mf5042sup3.mp4

Video S3. DOI: 10.1107/S2052252520007411/mf5042sup4.mp4

PDB reference: Structure of the light state of the LOV2 domain of phototropin-2 from *Arabidopsis thaliana*, 6s46

PDB reference: Structure of the dark state of the LOV2 domain of phototropin-2 from *Arabidopsis thaliana*, 6s45

## References

[bb1] Adams, P. D., Afonine, P. V., Bunkóczi, G., Chen, V. B., Davis, I. W., Echols, N., Headd, J. J., Hung, L.-W., Kapral, G. J., Grosse-Kunstleve, R. W., McCoy, A. J., Moriarty, N. W., Oeffner, R., Read, R. J., Richardson, D. C., Richardson, J. S., Terwilliger, T. C. & Zwart, P. H. (2010). *Acta Cryst.* D**66**, 213–221. 10.1107/S0907444909052925 PMC2815670 20124702

[bb2] Barends, T. R. M., Foucar, L., Ardevol, A., Nass, K., Aquila, A., Botha, S., Doak, R. B., Falahati, K., Hartmann, E., Hilpert, M., Heinz, M., Hoffmann, M. C., Köfinger, J., Koglin, J. E., Kovacsova, G., Liang, M., Milathianaki, D., Lemke, H. T., Reinstein, J., Roome, C. M., Shoeman, R. L., Williams, G. J., Burghardt, I., Hummer, G., Boutet, S. & Schlichting, I. (2015). *Science*, **350**, 445–450. 10.1126/science.aac5492 26359336

[bb3] Bourgeois, D. & Royant, A. (2005). *Curr. Opin. Struct. Biol.* **15**, 538–547. 10.1016/j.sbi.2005.08.002 16129597

[bb4] Broennimann, Ch., Eikenberry, E. F., Henrich, B., Horisberger, R., Huelsen, G., Pohl, E., Schmitt, B., Schulze-Briese, C., Suzuki, M., Tomizaki, T., Toyokawa, H. & Wagner, A. (2006). *J. Synchrotron Rad.* **13**, 120–130. 10.1107/S0909049505038665 16495612

[bb5] Casanas, A., Warshamanage, R., Finke, A. D., Panepucci, E., Olieric, V., Nöll, A., Tampé, R., Brandstetter, S., Förster, A., Mueller, M., Schulze-Briese, C., Bunk, O. & Wang, M. (2016). *Acta Cryst.* D**72**, 1036–1048. 10.1107/S2059798316012304 PMC5013597 27599736

[bb6] Chapman, H. N., Fromme, P., Barty, A., White, T. A., Kirian, R. A., Aquila, A., Hunter, M. S., Schulz, J., DePonte, D. P., Weierstall, U., Doak, R. B., Maia, F. R. N. C., Martin, A. V., Schlichting, I., Lomb, L., Coppola, N., Shoeman, R. L., Epp, S. W., Hartmann, R., Rolles, D., Rudenko, A., Foucar, L., Kimmel, N., Weidenspointner, G., Holl, P., Liang, M., Barthelmess, M., Caleman, C., Boutet, S., Bogan, M. J., Krzywinski, J., Bostedt, C., Bajt, S., Gumprecht, L., Rudek, B., Erk, B., Schmidt, C., Hömke, A., Reich, C., Pietschner, D., Strüder, L., Hauser, G., Gorke, H., Ullrich, J., Herrmann, S., Schaller, G., Schopper, F., Soltau, H., Kühnel, K. U., Messerschmidt, M., Bozek, J. D., Hau-Riege, S. P., Frank, M., Hampton, C. Y., Sierra, R. G., Starodub, D., Williams, G. J., Hajdu, J., Timneanu, N., Seibert, M. M., Andreasson, J., Rocker, A., Jönsson, O., Svenda, M., Stern, S., Nass, K., Andritschke, R., Schröter, C. D., Krasniqi, F., Bott, M., Schmidt, K. E., Wang, X., Grotjohann, I., Holton, J. M., Barends, T. R. M., Neutze, R., Marchesini, S., Fromme, R., Schorb, S., Rupp, D., Adolph, M., Gorkhover, T., Andersson, I., Hirsemann, H., Potdevin, G., Graafsma, H., Nilsson, B. & Spence, J. C. H. (2011). *Nature*, **470**, 73–77.

[bb7] Christie, J. M. (2007). *Annu. Rev. Plant Biol.* **58**, 21–45. 10.1146/annurev.arplant.58.032806.103951 17067285

[bb8] Emsley, P., Lohkamp, B., Scott, W. G. & Cowtan, K. (2010). *Acta Cryst.* D**66**, 486–501. 10.1107/S0907444910007493 PMC2852313 20383002

[bb9] Evans, P. R. (2011). *Acta Cryst.* D**67**, 282–292. 10.1107/S090744491003982X PMC3069743 21460446

[bb10] Evans, P. R. & Murshudov, G. N. (2013). *Acta Cryst.* D**69**, 1204–1214. 10.1107/S0907444913000061 PMC3689523 23793146

[bb11] Gotthard, G., Aumonier, S., De Sanctis, D., Leonard, G., von Stetten, D. & Royant, A. (2019). *IUCrJ*, **6**, 665–680. 10.1107/S205225251900616X PMC6608634 31316810

[bb12] Grünbein, M. L. & Nass Kovacs, G. (2019). *Acta Cryst.* D**75**, 178–191. 10.1107/S205979831801567X PMC6400261 30821706

[bb13] Hajdu, J., Wilmot, C. M., Neutze, R., Sjögren, T., Edman, K., Szöke, A. & Wilmouth, R. C. (2000). *Nat. Struct. Biol.* **7**, 1006–1012. 10.1038/80911 11062553

[bb14] Halavaty, A. S. & Moffat, K. (2007). *Biochemistry*, **46**, 14001–14009. 10.1021/bi701543e 18001137

[bb15] Halavaty, A. S. & Moffat, K. (2013). *Acta Cryst.* F**69**, 1316–1321. 10.1107/S1744309113029199 PMC3855711 24316821

[bb16] Harper, S. M., Neil, L. C. & Gardner, K. H. (2003). *Science*, **301**, 1541–1544. 10.1126/science.1086810 12970567

[bb17] Kabsch, W. (2010). *Acta Cryst.* D**66**, 125–132. 10.1107/S0907444909047337 PMC2815665 20124692

[bb18] Karplus, P. A. & Diederichs, K. (2012). *Science*, **336**, 1030–1033. 10.1126/science.1218231 PMC3457925 22628654

[bb19] Kennis, J. T. M., Crosson, S., Gauden, M., van Stokkum, I. H. M., Moffat, K. & van Grondelle, R. (2003). *Biochemistry*, **42**, 3385–3392. 10.1021/bi034022k 12653541

[bb20] Kern, J., Chatterjee, R., Young, I. D., Fuller, F. D., Lassalle, L., Ibrahim, M., Gul, S., Fransson, T., Brewster, A. S., Alonso-Mori, R., Hussein, R., Zhang, M., Douthit, L., de Lichtenberg, C., Cheah, M. H., Shevela, D., Wersig, J., Seuffert, I., Sokaras, D., Pastor, E., Weninger, C., Kroll, T., Sierra, R. G., Aller, P., Butryn, A., Orville, A. M., Liang, M., Batyuk, A., Koglin, J. E., Carbajo, S., Boutet, S., Moriarty, N. W., Holton, J. M., Dobbek, H., Adams, P. D., Bergmann, U., Sauter, N. K., Zouni, A., Messinger, J., Yano, J. & Yachandra, V. K. (2018). *Nature*, **563**, 421–425.

[bb21] Kottke, T., Heberle, J., Hehn, D., Dick, B. & Hegemann, P. (2003). *Biophys. J.* **84**, 1192–1201. 10.1016/S0006-3495(03)74933-9 PMC1302694 12547798

[bb22] Mehrabi, P., Schulz, E. C., Agthe, M., Horrell, S., Bourenkov, G., von Stetten, D., Leimkohl, J.-P., Schikora, H., Schneider, T. R., Pearson, A. R., Tellkamp, F. & Miller, R. J. D. (2019). *Nat. Methods*, **16**, 979–982. 10.1038/s41592-019-0553-1 31527838

[bb23] Mehrabi, P., Schulz, E. C., Dsouza, R., Müller-Werkmeister, H. M., Tellkamp, F., Miller, R. J. D. & Pai, E. F. (2019). *Science*, **365**, 1167–1170. 10.1126/science.aaw9904 31515393

[bb24] Nango, E., Royant, A., Kubo, M., Nakane, T., Wickstrand, C., Kimura, T., Tanaka, T., Tono, K., Song, C., Tanaka, R., Arima, T., Yamashita, A., Kobayashi, J., Hosaka, T., Mizohata, E., Nogly, P., Sugahara, M., Nam, D., Nomura, T., Shimamura, T., Im, D., Fujiwara, T., Yamanaka, Y., Jeon, B., Nishizawa, T., Oda, K., Fukuda, M., Andersson, R., Båth, P., Dods, R., Davidsson, J., Matsuoka, S., Kawatake, S., Murata, M., Nureki, O., Owada, S., Kameshima, T., Hatsui, T., Joti, Y., Schertler, G., Yabashi, M., Bondar, A.-N., Standfuss, J., Neutze, R. & Iwata, S. (2016). *Science*, **354**, 1552–1557. 10.1126/science.aah3497 28008064

[bb25] Okajima, K., Aihara, Y., Takayama, Y., Nakajima, M., Kashojiya, S., Hikima, T., Oroguchi, T., Kobayashi, A., Sekiguchi, Y., Yamamoto, M., Suzuki, T., Nagatani, A., Nakasako, M. & Tokutomi, S. (2014). *J. Biol. Chem.* **289**, 413–422. 10.1074/jbc.M113.515403 PMC3879564 24285544

[bb26] Pande, K., Hutchison, C. D. M., Groenhof, G., Aquila, A., Robinson, J. S., Tenboer, J., Basu, S., Boutet, S., DePonte, D. P., Liang, M., White, T. A., Zatsepin, N. A., Yefanov, O., Morozov, D., Oberthuer, D., Gati, C., Subramanian, G., James, D., Zhao, Y., Koralek, J., Brayshaw, J., Kupitz, C., Conrad, C., Roy-Chowdhury, S., Coe, J. D., Metz, M., Xavier, P. L., Grant, T. D., Koglin, J. E., Ketawala, G., Fromme, R., rajer, V., Henning, R., Spence, J. C., Ourmazd, A., Schwander, P., Weierstall, U., Frank, M., Fromme, P., Barty, A., Chapman, H. N., Moffat, K., van Thor, J. J. & Schmidt, M. (2016). *Science*, **352**, 725–729.

[bb27] Santoni, G., Zander, U., Mueller-Dieckmann, C., Leonard, G. & Popov, A. (2017). *J. Appl. Cryst.* **50**, 1844–1851. 10.1107/S1600576717015229 PMC5713145 29217993

[bb28] Schotte, F., Cho, H. S., Kaila, V. R. I., Kamikubo, H., Dashdorj, N., Henry, E. R., Graber, T. J., Henning, R., Wulff, M., Hummer, G., Kataoka, M. & Anfinrud, P. A. (2012). *Proc. Natl Acad. Sci. USA*, **109**, 19256–19261. 10.1073/pnas.1210938109 PMC3511082 23132943

[bb29] Schotte, F., Lim, M., Jackson, T. A., Smirnov, A. V., Soman, J., Olson, J. S., Phillips, G. N., Wulff, M. & Anfinrud, P. (2003). *Science*, **300**, 1944–1947. 10.1126/science.1078797 12817148

[bb30] Schulz, E. C., Mehrabi, P., Müller-Werkmeister, H. M., Tellkamp, F., Jha, A., Stuart, W., Persch, E., De Gasparo, R., Diederich, F., Pai, E. F. & Miller, R. J. D. (2018). *Nat. Methods*, **15**, 901–904. 10.1038/s41592-018-0180-2 30377366

[bb31] Šrajer, V. & Schmidt, M. (2017). *J. Phys. D Appl. Phys.* **50**, 373001. 10.1088/1361-6463/aa7d32 PMC5771432 29353938

[bb32] Stetten, D. von, Carpentier, P., Flot, D., Beteva, A., Caserotto, H., Dobias, F., Guijarro, M., Giraud, T., Lentini, M., McSweeney, S., Royant, A., Petitdemange, S., Sinoir, J., Surr, J., Svensson, O., Theveneau, P., Leonard, G. A. & Mueller-Dieckmann, C. (2020). *J. Synchrotron Rad.* **27**, 844–851. 10.1107/S1600577520004002 PMC7206554 32381789

[bb33] Stetten, D. von, Giraud, T., Carpentier, P., Sever, F., Terrien, M., Dobias, F., Juers, D. H., Flot, D., Mueller-Dieckmann, C., Leonard, G. A., De Sanctis, D. & Royant, A. (2015). *Acta Cryst.* D**71**, 15–26. 10.1107/S139900471401517X PMC4304682 25615856

[bb34] Swartz, T. E., Corchnoy, S. B., Christie, J. M., Lewis, J. W., Szundi, I., Briggs, W. R. & Bogomolni, R. A. (2001). *J. Biol. Chem.* **276**, 36493–36500. 10.1074/jbc.M103114200 11443119

[bb35] Theveneau, P., Baker, R., Barrett, R., Beteva, A., Bowler, M. W., Carpentier, P., Caserotto, H., Sanctis, D., Dobias, F., Flot, D., Guijarro, M., Giraud, T., Lentini, M., Leonard, G. A., Mattenet, M., McCarthy, A. A., McSweeney, S. M., Morawe, C., Nanao, M., Nurizzo, D., Ohlsson, S., Pernot, P., Popov, A. N., Round, A., Royant, A., Schmid, W., Snigirev, A., Surr, J. & Mueller-Dieckmann, C. (2013). *J. Phys. Conf. Ser.* **425**, 012001.

[bb36] Wheeler, M. J., Russi, S., Bowler, M. G. & Bowler, M. W. (2012). *Acta Cryst.* F**68**, 111–114. 10.1107/S1744309111054029 PMC3253849 22232186

[bb37] Wöhri, A. B., Katona, G., Johansson, L. C., Fritz, E., Malmerberg, E., Andersson, M., Vincent, J., Eklund, M., Cammarata, M., Wulff, M., Davidsson, J., Groenhof, G. & Neutze, R. (2010). *Science*, **328**, 630–633. 10.1126/science.1186159 20431017

