# Supplementary material for: Millisecond time-resolved serial oscillation crystallography of a blue-light photoreceptor at a synchrotron
Source: IUCrJ. 2020 Jun 24;7(Pt 4):728–36. doi: 10.1107/S2052252520007411 (PMC7340269; doi:10.1107/S2052252520007411)
Supplement: Supplementary file 1 [file m-07-00728-sup1.pdf]

IUCrJ

Volume 7 (2020)

Supporting information for article:

Millisecond time-resolved serial oscillation crystallography of a blue-light photoreceptor at a synchrotron

Sylvain Aumonier, Gianluca Santoni, Guillaume Gotthard, David von Stetten, Gordon A. Leonard and Antoine Royant

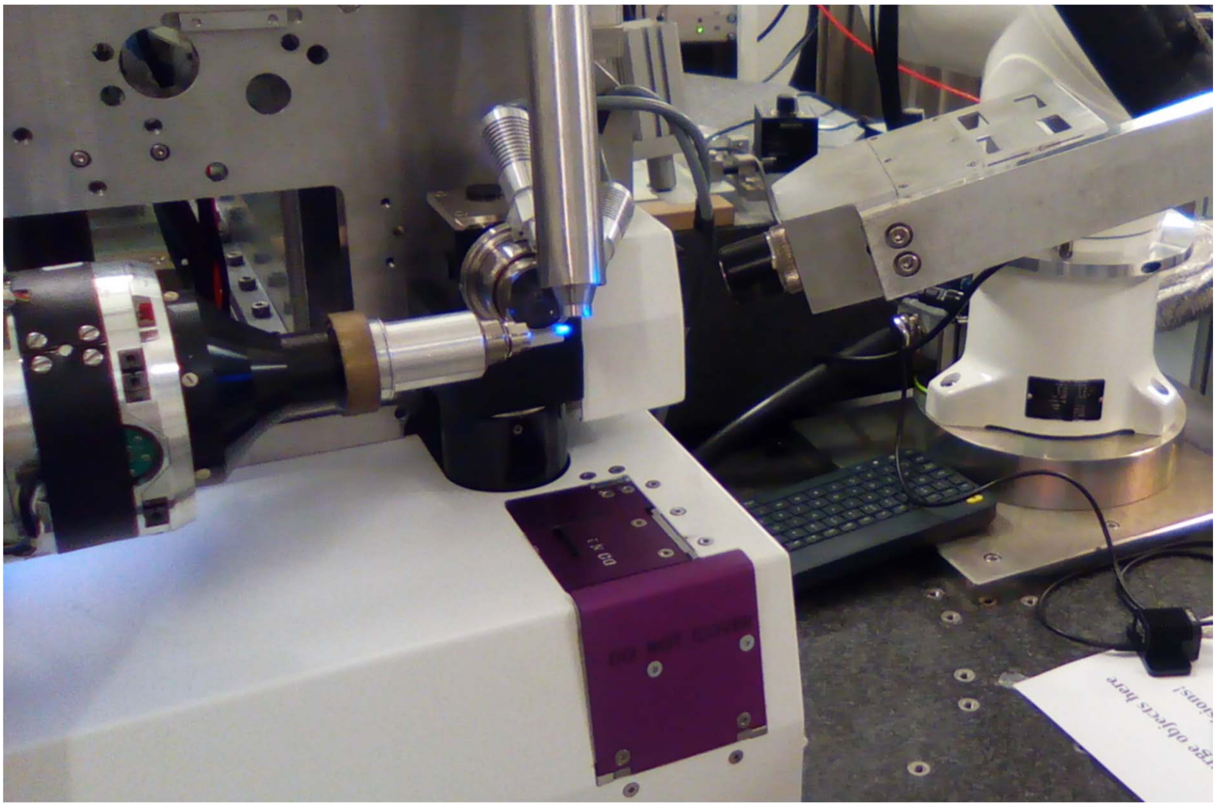

Figure S1 Experimental setup on beamline ID30A-3 of the ESRF. The crystal is maintained under the wet flux of an HC1 humidity controller (top) and is illuminated with a 470 nm LED (right).

(*a*)

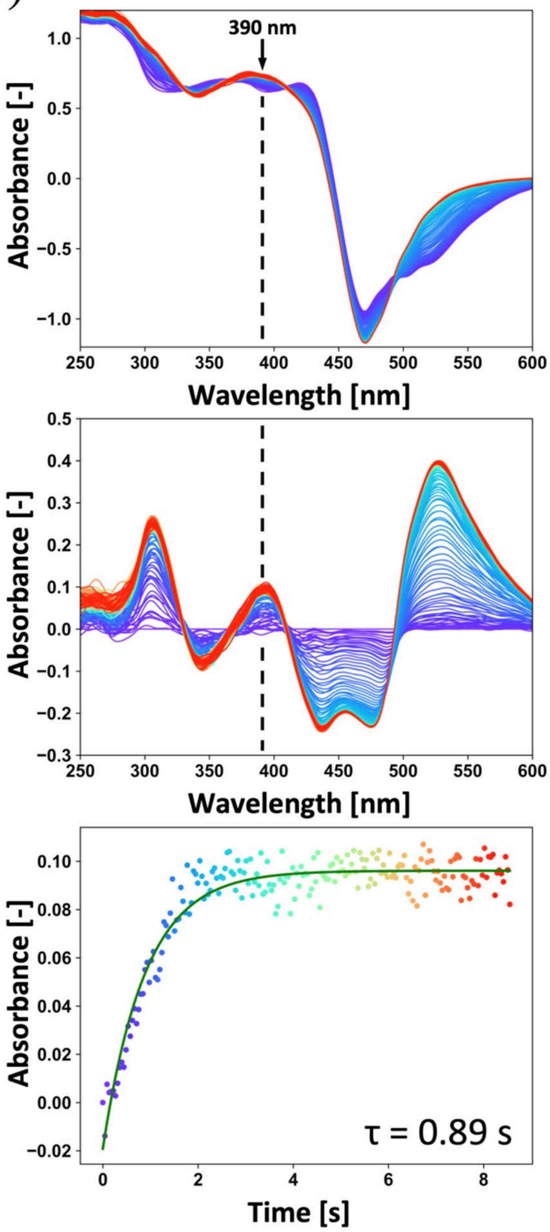
390 nm
Absorbance [-]
Wavelength [nm]
Absorbance [-]
Wavelength [nm]
Absorbance [-]
τ = 0.89 s
Time [s]

(*b*)

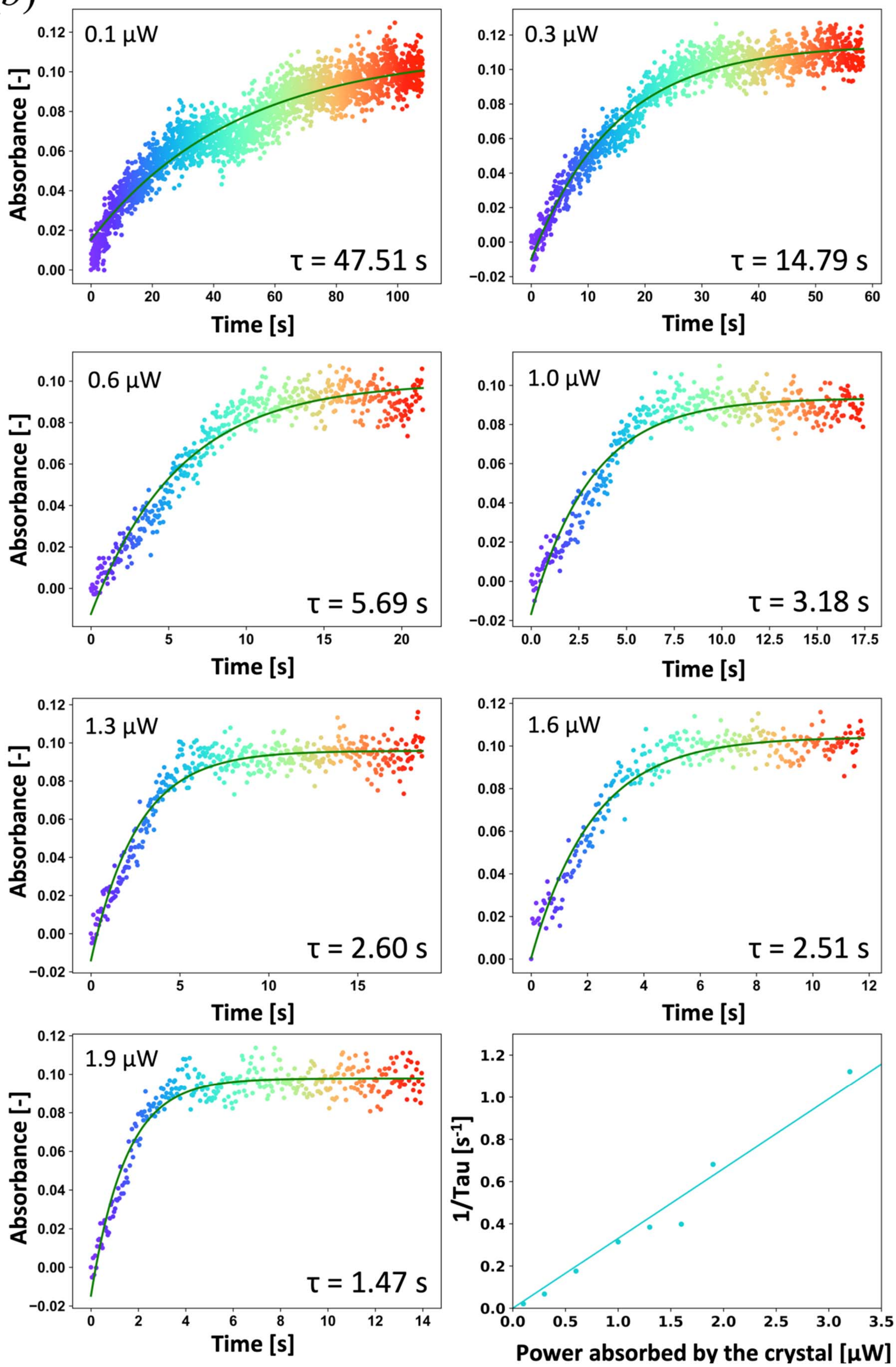

0.1 μW
τ = 47.51 s
0.3 μW
τ = 14.79 s
0.6 μW
τ = 5.69 s
1.0 μW
τ = 3.18 s
1.3 μW
τ = 2.60 s
1.6 μW
τ = 2.51 s
1.9 μW
τ = 1.47 s
Absorbance [-]
Time [s]
1/Tau [s-1]
Power absorbed by the crystal [μW]

Figure S2 Power titration of the speed of light state build-up in *At*Phot2LOV2 crystals. (*a*) From top to bottom: time series s(t) of UV-vis spectra of an *At*Phot2LOV2 crystal recorded at the *ic*OS Lab under illumination of a 470 nm LED with a nominal power of 5.0 mW, corresponding to an absorbed dose of 3.2 µW; time series [s(t) – s(t=0)] of difference spectra removing the contribution of the LED; evolution of the 390 nm peak, which is characteristic of light state build-up, modelled as a mono-exponential rise $a+b*\exp(-t/\tau)$, which approximates the linear rise reaching a plateau after ~2 times τ. (*b*) From left to right, top to bottom: evolution of the 390 nm peak for crystals under illumination of a 470 nm LED with an increasing nominal power of 0.1 to 3.0 mW. Bottom right panel: the $1/\tau$ *vs.* light power plot shows that the rate of light state build-up in crystals is linearly dependent on the number of photons per time unit.

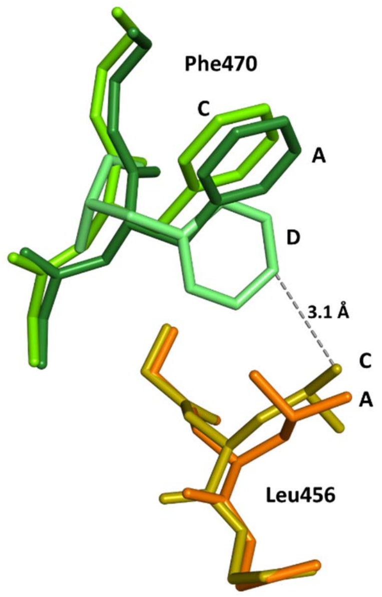

Figure S3 Reorientation and displacement of Leu456 triggered by the change in orientation of Phe470. The minor light state conformer D of Phe470 is orientated such that it forms a close van der Waals interaction with the side chain atoms of Leu456.

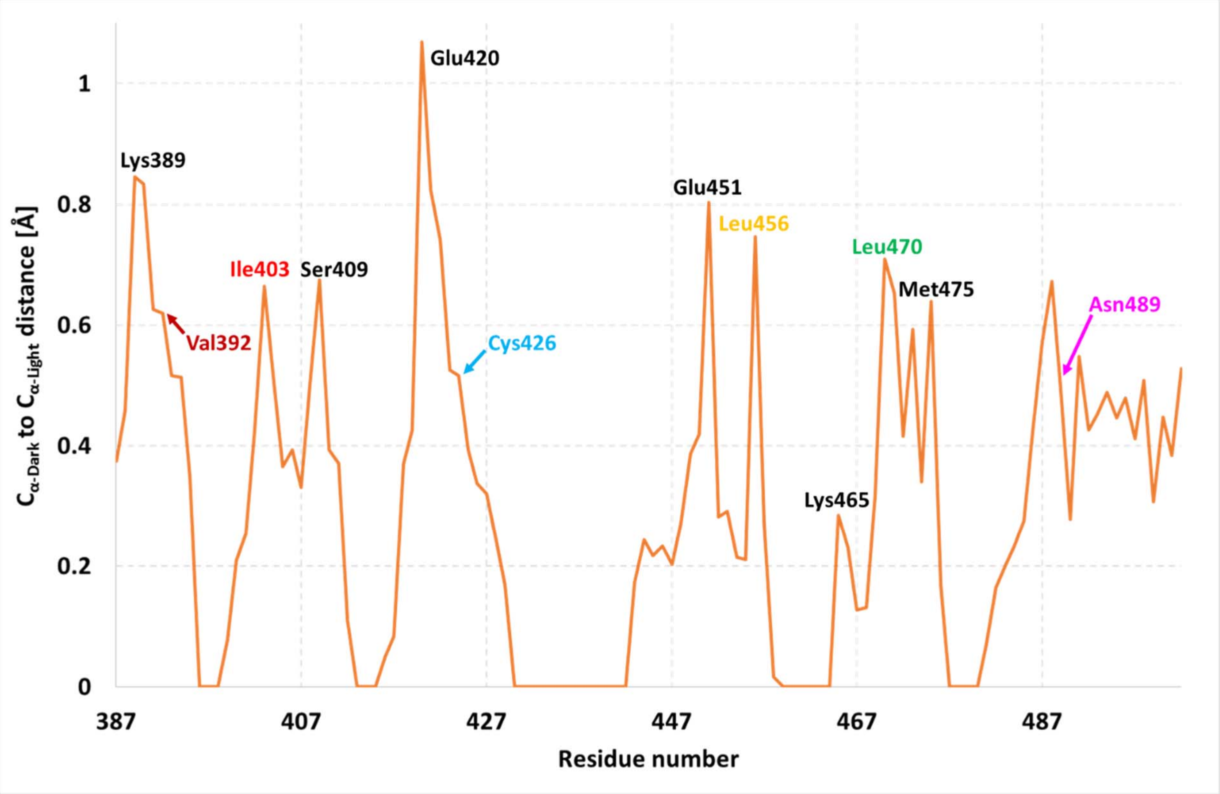

Figure S4 Cα to Cα distance plot between the dark and the light state structures. The plot highlights the magnitude of displacement of the various regions of the protein, and in particular of key residues (in colour).

Video S1 Propagation of light state build-up in an *At*Phot2LOV2 crystal upon LED irradiation coming from the top-right corner. The dark state absorbs between 400 and 490 nm and is thus fluorescent upon 470 nm excitation. The light state absorbs significantly between 350 and 450 nm, and is thus not fluorescent upon 470 nm excitation.

Video S2 Molecular movie of light state build-up in *At*Phot2LOV2 crystals with a 63 ms time resolution (view 1). Time evolution from 0 to 4158 ms of the $2F_{obs} - F_{calc}$ electron density map (contoured at a 1.2 σ level) showing the covalent bond formation between Cys426 and the FMN, disordering of the Gln489 side chain and displacement of the Phe470 side chain. The speed of the movie has been halved for visualization comfort (126 ms/frame). The dark state frame is paused for 800 ms to indicate clearly the start of the movie.

Video S3. Molecular movie of light state build-up in *At*Phot2LOV2 crystals with a 63 ms time resolution (view 2). Time evolution from 0 to 4158 ms of the $2F_{obs} - F_{calc}$ electron density map (contoured at a 0.7 σ level) showing the translation of Ile403 upon progressive disappearance of the B conformation of Cys426. The speed of the movie has been halved for visualization comfort (126 ms/frame). The dark state frame is paused for 800 ms to indicate clearly the start of the movie.

Table S1 Data reduction and refinement statistics for all 67 data sets.

Time point	Time [ms]	Integr. sub data sets	Retained sub data sets	Distance cutoff	Cell parameter a [Å]	Cell parameter c [Å]	Resolution range [Å]	Unique reflections	$CC_{1/2}$	$\langle I/\sigma(I)\rangle$	Multiplicity	R_{pim}	Completeness [%]	R_{cryst} [%]	R_{free} [%]	Bond length rmsd [Å]	Bond angle rmsd [°]
0	< 0	32	5	0.43	40.83	133.64	44.55-2.20 (2.27-2.20)	6273 (622)	0.999 (0.921)	14.0 (1.9)	87.3 (93.9)	0.051 (0.557)	100.0 (100.0)	20.2 (33.3)	24.9 (36.7)	0.002	0.55
1	63	50	26	0.39	40.73	133.28	44.43-2.45 (2.55-2.45)	4613 (500)	0.964 (0.775)	6.0 (2.8)	12.6 (13.0)	0.161 (0.795)	100.0 (100.0)	24.8 (38.2)	27.4 (0.3176)	0.003	0.65
2	126	52	38	0.41	40.77	133.45	44.48-2.50 (2.60-2.50)	4370 (478)	0.981 (0.812)	6.1 (2.9)	18.4 (19.1)	0.151 (0.813)	100.0 (100.0)	0.22.9 (29.7)	26.4 (32.4)	0.003	0.65
3	189	50	29	0.40	40.73	133.34	40.73-2.50 (2.60-2.50)	4365 (474)	0.979 (0.773)	6.7 (2.9)	14.0 (14.4)	0.151 (0.779)	100.0 (100.0)	23.0 (32.3)	25.0 (28.3)	0.003	0.65
4	252	48	39	0.45	40.77	133.38	40.77-2.60 (2.72-2.60)	3895 (461)	0.972 (0.739)	6.3 (2.7)	19.0 (19.9)	0.155 (0.899)	100.0 (100.0)	22.5 (30.8)	25.2 (29.7)	0.003	0.65
5	315	47	28	0.40	40.76	133.43	38.98-2.50 (2.60-2.50)	4369 (480)	0.967 (0.701)	5.4 (2.4)	13.6 (14.1)	0.189 (1.144)	100.0 (100.0)	23.4 (33.6)	26.6 (26.4)	0.003	0.65
6	378	44	27	0.45	40.76	133.48	44.49-2.55 (2.66-2.55)	4128 (471)	0.973 (0.720)	6.1 (2.6)	13.1 (13.7)	0.179 (0.912)	100.0 (100.0)	22.7 (31.9)	25.2 (28.9)	0.003	0.65
7	441	44	33	0.50	40.79	133.42	44.47-2.45 (2.55-2.45)	4639 (507)	0.965 (0.700)	5.1 (1.8)	16.0 (16.8)	0.242 (1.729)	100.0 (100.0)	22.9 (33.7)	25.6 (30.3)	0.003	0.65
8	504	42	33	0.52	40.74	133.16	44.39-2.45 (2.55-2.45)	4620 (502)	0.955 (0.718)	5.1 (1.8)	16.0 (16.5)	0.284 (2.092)	100.0 (100.0)	23.2 (36.6)	26.3 (36.5)	0.003	0.65
9	567	40	33	0.52	40.78	133.38	40.78-2.65 (2.78-2.65)	3708 (465)	0.970 (0.749)	5.7 (2.4)	15.9 (16.6)	0.167 (0.852)	100.0 (100.0)	21.7 (31.6)	24.7 (32.8)	0.003	0.65
10	630	41	36	0.51	40.80	133.46	39.02-2.65 (2.78-2.65)	3707 (461)	0.975 (0.776)	5.0 (1.8)	17.5 (18.5)	0.172 (1.091)	100.0 (100.0)	22.0 (33.7)	24.3 (36.8)	0.003	0.65
11	693	42	29	0.44	40.79	133.38	39.00-2.75 (2.90-2.75)	3320 (465)	0.971 (0.887)	6.2 (3.0)	13.9 (14.5)	0.138 (0.526)	99.9 (100.0)	21.5 (27.3)	23.7 (31.9)	0.003	0.65
12	756	44	26	0.44	40.78	133.35	39.00-2.75 (2.90-2.75)	3320 (465)	0.966 (0.770)	5.5 (2.6)	12.6 (13.1)	0.153 (0.652)	99.9 (99.9)	22.1 (30.0)	25.7 (32.7)	0.003	0.65
13	819	45	43	0.56	40.84	133.41	44.47-2.45 (2.55-2.45)	4642 (498)	0.982 (0.743)	7.0 (1.8)	41.8 (43.2)	0.238 (2.386)	100.0 (100.0)	24.3 (40.3)	28.6 (42.2)	0.003	0.65
14	882	44	38	0.50	40.82	133.32	44.44-2.70 (2.83-2.70)	3518 (433)	0.968 (0.734)	4.9 (1.5)	18.3 (19.1)	0.165 (1.012)	100.0 (100.0)	22.4 (34.4)	25.3 (35.0)	0.003	0.65
15	945	42	34	0.53	40.81	133.36	40.81-2.75 (2.90-2.75)	3310 (469)	0.969 (0.764)	5.4 (2.3)	16.5 (17.2)	0.164 (0.756)	100.0 (100.0)	22.5 (31.9)	27.3 (43.9)	0.003	0.65

Time point	Time [ms]	Integr. sub data sets	Retained sub data sets	Distance cutoff	Cell parameter a [Å]	Cell parameter c [Å]	Resolution range [Å]	Unique reflections	$CC_{1/2}$	<I/σ(I)>	Multiplicity	R_{pim}	Completeness [%]	R_{cryst} [%]	R_{free} [%]	Bond length rmsd [Å]	Bond angle rmsd [°]
16	1008	42	35	0.53	40.84	133.36	44.45-2.75 (2.90-2.75)	3328 (468)	0.972 (0.863)	5.5 (2.1)	16.8 (17.8)	0.170 (0.770)	100.0 (100.0)	22.6 (30.9)	25.1 (28.9)	0.003	0.65
17	1071	43	36	0.60	40.85	133.28	44.43-2.75 (2.90-2.75)	3333 (467)	0.965 (0.841)	5.1 (2.1)	17.3 (18.0)	0.178 (0.878)	100.0 (100.0)	21.7 (33.1)	26.3 (30.1)	0.003	0.65
18	1134	41	31	0.53	40.80	133.18	40.80-2.75 (2.90-2.75)	3321 (466)	0.963 (0.811)	5.3 (2.3)	14.9 (15.5)	0.185 (0.875)	100.0 (100.0)	22.6 (31.6)	26.2 (33.3)	0.003	0.65
19	1197	46	34	0.55	40.87	133.31	44.44-2.65 (2.78-2.65)	3724 (466)	0.966 (0.710)	4.6 (1.4)	16.4 (17.0)	0.202 (1.392)	100.0 (100.0)	22.9 (35.5)	26.7 (23.1)	0.003	0.65
20	1260	41	30	0.52	40.83	133.24	44.41-2.75 (2.90-2.75)	3334 (469)	0.962 (0.724)	5.4 (2.4)	14.3 (14.9)	0.187 (0.787)	100.0 (100.0)	22.3 (30.2)	26.8 (35.0)	0.003	0.65
21	1323	42	32	0.51	40.84	133.19	44.40-2.65 (2.78-2.65)	3708 (464)	0.964 (0.790)	4.9 (1.5)	15.5 (16.2)	0.239 (1.697)	100.0 (100.0)	22.7 (32.2)	26.4 (32.2)	0.003	0.65
22	1386	44	33	0.58	40.84	133.33	44.44-2.75 (2.90-2.75)	3340 (469)	0.970 (0.709)	4.8 (1.9)	15.9 (16.5)	0.177 (0.814)	100.0 (100.0)	22.7 (29.8)	26.4 (36.0)	0.003	0.65
23	1449	46	38	0.61	40.91	133.40	44.47-2.75 (2.90-2.75)	3347 (466)	0.970 (0.837)	4.8 (1.8)	18.2 (19.0)	0.186 (0.875)	100.0 (100.0)	22.6 (32.0)	26.2 (42.7)	0.003	0.65
24	1512	46	36	0.66	40.85	133.35	40.85-2.90 (3.08-2.90)	2870 (423)	0.971 (0.707)	5.4 (2.5)	17.0 (17.3)	0.156 (0.680)	100.0 (100.0)	21.5 (26.2)	23.6 (23.0)	0.003	0.65
25	1575	44	38	0.67	40.90	133.31	44.44-2.75 (2.90-2.75)	3348 (469)	0.968 (0.754)	5.2 (2.1)	18.1 (18.9)	0.194 (0.878)	100.0 (100.0)	22.8 (30.2)	26.4 (37.6)	0.003	0.65
26	1638	43	37	0.70	40.91	133.37	44.46-2.65 (2.78-2.65)	3726 (466)	0.957 (0.716)	4.5 (1.5)	17.7 (18.4)	0.248 (1.655)	100.0 (100.0)	22.8 (31.6)	25.6 (26.1)	0.003	0.65
27	1701	45	34	0.71	40.91	133.30	44.43-2.75 (2.90-2.75)	335 (471)	0.957 (0.811)	4.9 (2.0)	16.3 (16.9)	0.204 (0.844)	100.0 (100.0)	22.9 (29.7)	26.7 (31.0)	0.003	0.65
28	1764	47	35	0.61	40.91	133.41	40.91-2.70 (2.83-2.70)	3515 (434)	0.948 (0.708)	4.7 (1.6)	17.0 (17.5)	0.247 (1.537)	100.0 (100.0)	23.5 (30.4)	26.4 (34.0)	0.003	0.65
29	1827	42	37	0.66	40.88	133.46	40.88-2.70 (2.83-2.70)	3521 (435)	0.962 (0.702)	4.5 (1.5)	17.9 (18.8)	0.217 (1.301)	100.0 (100.0)	23.5 (33.0)	27.9 (39.9)	0.003	0.65
30	1890	45	37	0.71	40.91	133.33	44.44-2.60 (2.72-2.60)	3940 (461)	0.959 (0.704)	4.3 (1.2)	18.0 (19.0)	0.311 (2.235)	100.0 (100.0)	23.6 (30.2)	27.5 (43.3)	0.003	0.65
31	1953	45	25	0.59	40.88	133.48	44.49-2.75 (2.90-2.75)	3344 (460)	0.946 (0.774)	4.5 (1.8)	11.9 (12.4)	0.208 (0.976)	100.0 (100.0)	23.3 (30.5)	26.6 (30.1)	0.003	0.65
32	2016	42	32	0.55	40.89	133.46	40.89-2.75 (2.90-2.75)	3346 (463)	0.940 (0.810)	5.1 (2.0)	15.3 (15.7)	0.213 (0.871)	100.0 (100.0)	23.0 (33.5)	28.2 (40.6)	0.003	0.65

Time point	Time [ms]	Integr. sub data sets	Retained sub data sets	Distance cutoff	Cell parameter a [Å]	Cell parameter c [Å]	Resolution range [Å]	Unique reflections	$CC_{1/2}$	<I/σ(I)>	Multiplicity	R_{pim}	Completeness [%]	R_{cryst} [%]	R_{free} [%]	Bond length rmsd [Å]	Bond angle rmsd [°]
33	2079	40	34	0.63	40.92	133.35	44.45-2.75 (2.90-2.75)	3358 (472)	0.949 (0.720)	6.9 (1.7)	15.8 (16.5)	0.232 (1.164)	100.0 (100.0)	23.3 (30.5)	26.1 (47.2)	0.003	0.65
34	2142	45	40	0.67	40.95	133.37	44.46-2.75 (2.90-2.75)	3361 (468)	0.956 (0.772)	5.1 (1.8)	18.0 (18.8)	0.210 (1.095)	100.0 (100.0)	23.3 (31.3)	27.0 (32.9)	0.003	0.65
35	2205	39	37	0.62	40.98	133.45	44.48-2.75 (2.90-2.75)	3366 (469)	0.973 (0.846)	4.5 (1.1)	17.3 (18.0)	0.159 (0.927)	100.0 (100.0)	23.4 (32.8)	26.7 (38.2)	0.003	0.65
36	2268	43	36	0.72	40.99	133.49	44.50-2.75 (2.90-2.75)	3366 (469)	0.976 (0.798)	4.4 (1.0)	16.9 (17.8)	0.162 (0.959)	100.0 (100.0)	24.0 (37.6)	26.2 (48.4)	0.003	0.65
37	2331	41	40	0.69	40.96	133.38	44.46-2.75 (2.90-2.75)	3357 (468)	0.967 (0.698)	4.6 (1.2)	19.0 (19.6)	0.160 (0.961)	100.0 (100.0)	24.0 (34.1)	28.8 (53.0)	0.003	0.65
38	2394	42	37	0.77	40.98	133.38	40.98-2.75 (2.90-2.75)	3361(469)	0.979 (0.802)	4.5 (1.2)	17.4 (18.2)	0.151 (0.885)	100.0 (100.0)	24.1 (32.8)	27.0 (43.4)	0.003	0.65
39	2457	42	40	0.78	40.99	133.40	40.99-2.75 (2.90-2.75)	3355 (469)	0.976 (0.806)	4.5 (1.2)	18.8 (19.5)	0.159 (0.885)	100.0 (100.0)	24.1 (34.2)	28.0 (41.8)	0.003	0.65
40	2520	42	38	0.66	40.96	133.29	44.43-2.75 (2.90-2.75)	3346 (471)	0.979 (0.809)	4.5 (1.2)	18.0 (18.8)	0.154 (0.850)	100.0 (100.0)	23.9 (35.7)	27.4 (37.6)	0.003	0.65
41	2583	41	36	0.72	40.98	133.40	40.98-2.75 (2.90-2.75)	3364 (469)	0.910 (0.744)	4.5 (1.7)	16.8 (17.4)	0.308 (0.971)	100.0 (100.0)	25.3 (30.8)	28.6 (37.4)	0.003	0.65
42	2646	39	33	0.74	41.01	133.41	41.01-2.75 (2.90-2.75)	3365 (467)	0.970 (0.762)	4.3 (1.3)	15.4 (16.1)	0.167 (0.879)	100.0 (100.0)	24.4 (33.5)	29.1 (44.4)	0.003	0.65
43	2709	40	36	0.71	41.01	133.44	41.01-2.75 (2.90-2.75)	3365 (469)	0.976 (0.940)	4.4 (1.2)	17.4 (16.9)	0.163 (0.905)	100.0 (100.0)	24.0 (33.4)	28.4 (39.1)	0.003	0.65
44	2772	43	40	0.92	41.00	133.37	44.46-2.75 (2.90-2.75)	3366 (467)	0.818 (0.819)	4.5 (1.3)	18.8 (19.5)	0.205 (1.314)	100.0 (100.0)	24.1 (31.8)	30.1 (39.1)	0.003	0.65
45	2835	47	39	0.73	41.01	133.35	44.45-2.80 (2.95-2.80)	3208 (454)	0.975 (0.764)	4.6 (1.5)	18.2 (18.9)	0.179 (1.009)	100.0 (100.0)	24.0 (32.8)	26.7 (28.8)	0.003	0.65
46	2898	46	37	0.67	40.97	133.34	44.45-2.75 (2.90-2.75)	3361 (472)	0.969 (0.734)	4.5 (1.2)	17.2 (17.7)	0.165 (0.954)	100.0 (100.0)	23.7 (33.6)	27.1 (36.1)	0.003	0.65
47	2961	46	39	0.71	40.96	133.22	44.41-2.75 (2.90-2.75)	3348 (474)	0.971 (0.831)	4.5 (1.3)	18.8 (19.3)	0.181 (1.095)	100.0 (100.0)	24.2 (31.9)	26.4 (40.0)	0.003	0.65
48	3024	43	23	0.55	40.85	133.31	40.85-2.80 (2.95-2.80)	3179 (448)	0.963 (0.714)	5.1 (1.9)	10.5 (10.7)	0.173 (0.894)	100.0 (100.0)	23.2 (29.9)	27.0 (34.8)	0.003	0.65

Time point	Time [ms]	Integr. sub data sets	Retained sub data sets	Distance cutoff	Cell parameter a [Å]	Cell parameter c [Å]	Resolution range [Å]	Unique reflections	$CC_{1/2}$	<I/σ(I)>	Multiplicity	R_{pim}	Completeness [%]	R_{cryst} [%]	R_{free} [%]	Bond length rmsd [Å]	Bond angle rmsd [°]
49	3087	43	36	0.73	40.99	133.36	40.99-2.75 (2.90-2.75)	3364 (472)	0.799 (0.747)	5.5 (1.9)	16.6 (17.1)	0.558 (1.088)	100.0 (100.0)	26.7 (29.4)	26.8 (38.7)	0.003	0.65
50	3150	43	37	0.68	40.94	133.36	44.45-2.80 (2.95-2.80)	3195 (451)	0.969 (0.760)	4.7 (1.4)	17.2 (18.0)	0.172 (1.073)	100.0 (100.0)	23.5 (29.7)	27.4 (33.6)	0.003	0.65
51	3213	43	34	0.69	40.93	133.27	44.42-2.75 (2.90-2.75)	3353 (474)	0.846 (0.798)	4.1 (1.4)	15.9 (16.6)	0.401 (0.930)	100.0 (100.0)	25.3 (31.7)	29.2 (29.6)	0.003	0.65
52	3276	41	32	0.60	40.93	133.36	39.13-2.95 (3.13-2.95)	2729 (413)	0.930 (0.716)	5.3 (2.3)	14.8 (15.0)	0.207 (0.709)	99.9 (100.0)	23.6 (29.7)	26.2 (30.0)	0.003	0.65
53	3339	40	35	0.77	40.95	133.32	40.95-2.75 (2.90-2.75)	3352 (473)	0.938 (0.738)	4.5 (1.5)	16.4 (16.8)	0.213 (0.976)	100.0 (100.0)	24.3 (31.9)	29.3 (38.8)	0.003	0.65
54	3402	36	28	0.64	41.01	133.35	44.45-2.75 (2.90-2.75)	3362 (467)	0.955 (0.700)	4.7 (1.7)	13.0 (13.6)	0.203 (0.959)	100.0 (100.0)	24.4 (32.1)	27.7 (39.2)	0.003	0.65
55	3456	37	33	0.74	40.98	133.30	44.43-2.75 (2.90-2.75)	3363 (475)	0.938 (0.803)	4.7 (1.6)	15.4 (16.0)	0.189 (0.976)	100.0 (100.0)	24.4 (33.2)	27.4 (37.8)	0.003	0.65
56	3528	40	30	0.64	40.97	133.29	44.43-2.75 (2.90-2.75)	3363 (475)	0.969 (0.775)	4.9 (1.8)	14.0 (14.4)	0.203 (0.979)	100.0 (100.0)	24.8 (32.8)	28.3 (38.3)	0.003	0.65
57	3591	38	31	0.64	40.98	133.33	44.44-2.75 (2.90-2.75)	3365 (472)	0.872 (0.734)	4.8 (1.5)	14.4 (15.0)	0.347 (1.118)	100.0 (100.0)	24.7 (31.1)	29.1 (43.7)	0.003	0.65
58	3654	41	36	0.74	40.99	133.31	39-18-2.75 (2.90-2.75)	3359 (472)	0.975 (0.769)	4.5 (1.1)	17.0 (17.8)	0.165 (0.982)	100.0 (100.0)	24.3 (34.6)	30.1 (39.0)	0.003	0.65
59	3717	40	36	0.66	40.97	133.26	44.42-2.75 (2.90-2.75)	3351 (474)	0.958 (0.750)	5.0 (1.6)	16.9 (17.8)	0.200 (1.068)	100.0 (100.0)	24.4 (33.4)	28.3 (37.2)	0.003	0.65
60	3780	40	35	0.69	40.98	133.23	34.90-2.75 (2.90-2.75)	3357 (475)	0.946 (0.719)	4.5 (1.2)	16.5 (17.3)	0.174 (0.969)	100.0 (100.0)	24.4 (37.8)	28.9 (42.3)	0.003	0.65
61	3843	39	35	0.67	40.98	133.31	44.44-2.85 (3.00-2.85)	3043 (412)	0.970 (0.749)	4.7 (1.3)	16.3 (16.9)	0.142 (0.710)	100.0 (100.0)	24.0 (32.1)	27.5 (45.4)	0.003	0.65
62	3906	41	38	0.70	40.97	133.22	44.41-2.75 (2.90-2.75)	3361 (475)	0.975 (0.753)	4.5 (1.3)	17.8 (18.4)	0.182 (1.070)	100.0 (100.0)	24.6 (34.0)	28.4 (38.9)	0.003	0.65
63	3969	38	25	0.64	40.87	133.21	39.07-2.90 (3.08-2.90)	2853 (425)	0.953 (0.708)	5.0 (1.6)	11.6 (12.0)	0.158 (0.672)	99.9 (100.0)	23.4 (28.9)	28.4 (36.7)	0.003	0.65
64	4032	44	41	0.70	41.01	133.28	44.43-2.80 (2.95-2.80)	3195 (454)	0.979 (0.747)	4.9 (1.3)	19.2 (19.9)	0.147 (0.848)	100.0 (100.0)	24.1 (31.1)	27.4 (28.6)	0.003	0.65
65	4095	40	35	0.69	41.03	133.32	44.44-2.75 (2.90-2.75)	3364 (467)	0.972 (0.791)	4.5 (1.1)	16.4 (17.2)	0.163 (0.954)	100.0 (100.0)	24.7 (34.1)	28.3 (35.3)	0.003	0.65
66	4158	36	32	0.65	41.01	133.15	41.01-2.75 (2.90-2.75)	3355 (468)	0.974 (0.766)	4.5 (1.2)	15.0 (15.8)	0.146 (0.766)	100.0 (100.0)	24.2 (32.9)	29.2 (37.9)	0.003	0.65

Table S2 Rise and decay time constants of key residue population evolutions derived from structural analysis.

Residue (conformation)	Time constant [ms]
FMN (A,C)	1325
Val392 (A,C)	1490
Ile403 (A,C)	1527
Cys426 (A)	1546
Cys426 (B)	1185
Cys 426 (C)	1434
Leu456 (A, C)	1527
Phe470 (A)	1360
Phe470 (C)	1304
Phe470 (D)	1576
Gln489 (A, C)	1621
Average	1445 +/- 135
